# Demographic changes in COVID-19 mortality during the pandemic: analysis of trends in disparities among workers using California’s mortality surveillance system

**DOI:** 10.1186/s12889-024-19257-4

**Published:** 2024-07-09

**Authors:** Elisabeth Gebreegziabher, David Bui, Kristin J. Cummings, Matthew Frederick, Alyssa Nguyen, Caroline Collins, David Melton, Alice Yang, Seema Jain, Ximena Vergara

**Affiliations:** 1 Occupational Health Branch, California Department of Public Health, 850 Marina Bay Parkway, Richmond, CA 94804 USA; 2 Heluna Health, 13300 Crossroads Pkwy. N #450, City of Industry, CA 91746 USA; 3 Infectious Diseases Branch, California Department of Public Health, Richmond, CA 94804 USA; 4 Public Health Institute, Oakland, CA 94607 USA

**Keywords:** COVID-19, Mortality, Disparities, Workers, Demographics, Trends

## Abstract

**Background:**

There is limited information on the extent and patterns of disparities in COVID-19 mortality throughout the pandemic. We aimed to examine trends in disparities by demographics over variants in the pre- and post-vaccine availability period among Californian workers using a social determinants of health lens.

**Methods:**

Using death certificates, we identified all COVID-19 deaths that occurred between January 2020 and May 2022 among workers aged 18–64 years in California (CA). We derived estimates for at-risk worker populations using the Current Population Survey. The waves of COVID-19 mortality in the pre-vaccine availability period were March 2020-June 2020 (wave 1), and July 2020-November 2020 (wave 2), and in the post-vaccine availability period: December 2020-May 2021 (wave 3), June 2021-January 2022 (wave 4), and February 2022-May 2022 (wave 5). Poisson regression models with robust standard errors were used to determine wave-specific mortality rate ratios (MRRs). We examined the change in MRR across waves by including an interaction term between each demographic characteristic and wave period in different models. The role of potential misclassification of Race/ethnicity on death certificates was examined using probabilistic quantitative bias analysis as sensitivity analysis.

**Results:**

Among the 24.1 million working age CA population included in the study, there were 26,068 COVID-19 deaths in the period between January 2020 and May 2022. Compared with their respective reference groups, workers who were 50–64 years old, male, Native Hawaiian, Latino, or African American, foreign-born; individuals who had lower education; and unmarried were disproportionately affected by COVID-19 mortality. While disparities by sex, race/ethnicity and foreign-born status narrowed in later waves (post-vaccine availability), disparities by age, education level and marital status did not change substantially across waves.

**Conclusion:**

Demographic disparities in COVID-19 mortality narrowed in the post-vaccine availability waves. However, the existence of disparities across all waves of the pandemic, even in an era of widespread vaccine coverage, could indicate remaining gaps in prevention and differential vulnerability. Addressing the underlying social, structural, and occupational factors that contribute to these disparities is critical for achieving health equity.

**Supplementary Information:**

The online version contains supplementary material available at 10.1186/s12889-024-19257-4.

## Introduction

During the pandemic, COVID-19 was among the top causes of death in the U.S [1]. Excess COVID-19 mortality was detected among older individuals [2], men [3, 4], individuals without college or high school diplomas [5–7], foreign-born persons [8–10], individuals with non-Hispanic black and Latino race/ethnicity [11–13], and frontline workers [14]. Moreover, nearly one-quarter of COVID-19 cases are attributed to work, which makes the intersection of work and other social determinants of health critical [15, 16].

Social determinants of health (SDOH) capture the conditions in which people live and work, strongly shape health-related behaviors and outcomes, and are increasingly recognized for their role in health disparities [17, 18]. Previous studies show that SDOH are often the underlying drivers of disparities in health outcomes and may be targeted for change as a mechanism to reduce disparities in outcomes such as COVID-19 [19, 20]. For instance, individual COVID-19 preventative health behaviors, such as mask wearing, social distancing, sheltering in place, and vaccine uptake, are embedded within social health literacy, social networks and work [5, 21]. The ability to telework, access to sick leave and protection through COVID-19 safety and mitigation policies reduced adverse COVID-19 outcomes [22].

Understanding when and which workers died from COVID-19 has important implications for future prevention. While there is some evidence regarding disparities in COVID-19 mortality according to demographics, a comprehensive assessment of worker characteristics can provide insights into how several demographic risk factors could interact and help identify subgroups of worker populations that were disproportionately impacted by COVID-19 [23]. In addition, research on how disparities by worker demographics have changed throughout the pandemic within the context of changes in policies, resources, mitigation measures and the availability of vaccines is scarce. An assessment of patterns in COVID-19 mortality among workers can help identify and characterize disparities that have persisted throughout the pandemic.

The objective of this study was to examine disparities in the COVID-19 mortality rate among working Californians by sex, race/ethnicity, age, education level, foreign-born status and marital status and temporal trends in disparities over variant periods or waves of mortality.

## Methods

### Study design and population

For this population-based retrospective cohort study, we used data from California’s COVID-19 case registry, the Electronic Death Registration System (EDRS), and the Employment Development Department (EDD) to identify all COVID-19 deaths among individuals aged 18–64 years who were in the labor force. We used current population survey (CPS) data to derive estimates of the California population (aged 18–64 years) that was in the labor force during the various waves of COVID-19 mortality between January 2020 and May 2022.

We defined the waves based on the peaks of COVID-19 mortality [24, 25]. The dominant variants circulating during these waves were Alpha and Epsilon in wave 3, Delta in wave 4 and Omicron during wave 5 [26, 27]. The five waves included the periods of March 2020-June 2020 (Wave 1), and July 2020-November 2020 (Wave 2), in the pre-vaccination period, and December 2020-May 2021 (Wave 3/ Epsilon & Alpha variants), June 2021-January 2022 (Wave 4/Delta variant), and February 2022-May 2022 (Wave 5/Omicron variant) in the post vaccine availability period given that COVID-19 vaccines were first available in December 2020 [28]. Based on the date of death reported in the death certificates, COVID-19 deaths were attributed to a specific wave.

### Data collection and measures

#### Data source

The mortality data, including worker demographic characteristics such as age, sex, race/ethnicity, education level, foreign-born status, and marital status, were obtained from the electronic death certificates within the EDRS. The California COVID-19 case registry, which contains local health jurisdiction reports of all laboratory-confirmed COVID-19 cases among California residents and case patient vital status, was used to identify COVID-19 worker deaths. EDRS and EDD records derived from quarterly tax reports were used to determine individuals’ employment status prior to death. Variables from the three data sources, EDRS, the COVID-19 case registry and the EDD, were merged to generate a dataset of the COVID-19 decedents and their characteristics. Additional details on definitions and data source linkages were described in previous studies [12, 29].

The denominators used to estimate the California population of workers at risk of death during each COVID-19 mortality wave were obtained from the monthly CPS, which is a US household survey conducted jointly by the US Census Bureau and the Bureau of Labor Statistics. We used CPS estimates available from the Integrated Public Use Microdata Series (IPUMS) website to obtain person-level data with strata of the same set of characteristics as the mortality data and weights corresponding to the number of individuals represented [30]. After restricting the sample to individuals aged 18–64 years and in the labor force, population counts were created by summing CPS person weights for each wave to account for seasonal variation in the worker population. We constructed a dataset of COVID-19 decedents who were confirmed or likely working and working populations at risk by appending the CPS population dataset to the mortality dataset.

The California Health and Human Services Agency’s Committee for the Protection of Human Subjects determined that this project was exempt from review because the activities involved public health practice/surveillance, not research. Informed consent for the use of identifiable information by a public health agency for public health practice/surveillance is not needed.

#### Measures

The demographic characteristics of the workers (exposures) analyzed, include age group, sex, race/ethnicity, foreign-born status, education level, and marital status. The age groups included 18–29 (reference group), 30–39, 40–49, 50–59 and 60–64 years. Race/ethnicity included non-Hispanic White (reference group, hereafter referred as white), non-Hispanic Black or African American (hereafter referred as African American), non-Hispanic Asian American (hereafter referred as Asian), non-Hispanic American Indian or Alaska Native (hereafter referred as American Indian), non-Hispanic Native Hawaiian or Other Pacific Islander (hereafter referred as Native Hawaiian), and Hispanic or Latino (hereafter referred as Latino). Education level was categorized as follows: 1) high school or less (including GED, high school graduate and no diploma, referent group); 2) bachelor’s degree, associate degree, or some college group; 3) master’s degree; 4) professional; or 5) doctorate. Sex, foreign-born status, and marital status were binary with female, U.S. birth (including U.S territory) and currently married status used as reference groups, respectively. In each analysis, groups with lower or the lowest mortality rates in most waves were chosen as the reference group, except for education level, for which the group with the largest sample size (fatalities) was chosen as the reference group (i.e., education level of high school or less).

The outcome was COVID-19 mortality based on local health jurisdiction determinations reported in the California COVID-19 case registry. All other deaths were considered non-COVID-19 deaths. The worker population strata obtained from the CPS represented living individuals.

#### Statistical analysis

We used Poisson regression with robust standard errors to generate mortality rate ratios (MRRs) for each characteristic compared to those of the reference group. Using complete-case analysis, we ran separate models for each wave using wave-specific weights and a specific covariate adjustment set (for each main predictor) in the regression models. Using separate models for each characteristic, we assessed the significance of the change in MRR across waves by including an interaction term between each characteristic and wave period [31]. Comparisons were made by selecting the previous wave period as the referent period; e.g., changes from Waves 1 (ref) to 2, 2 (ref) to 3, 3 (ref) to 4 and 4 (ref) to 5 were examined. Results were considered statistically significant at an alpha level of p < 0.05. To adjust for multiple hypothesis testing, we used the Romano-wolf method, which uses bootstrap resampling to control for the familywise error rate and has been shown more powerful in its ability to correctly reject false null hypotheses than prior methods [32].

We used directed acyclic graphs available here as a conceptual framework to help determine the relationship between variables and covariates to adjust for in each analysis. The adjustment set for each of the six exposures analyzed were as follows: 1) for analysis by age group: sex; 2) for analysis by education level: foreign-born status, race/ethnicity, age group, and sex; 3) for analysis by race/ethnicity: age group, sex, and foreign-born status; 4) for analysis by sex: age group; 5) for analysis by marital status: age group, sex, and race/ethnicity; and 6) for analysis by foreign-born status: age group and sex.

#### Sensitivity analyses

We undertook several sensitivity analyses to examine the role of misclassification of race/ethnicity, misclassification of education level and the impacts of restriction by employment status on our results.

Given previous reports of misclassification of race/ethnicity on death certificates [33, 34], we conducted a quantitative bias analysis to estimate the sensitivity of our findings to potential misclassification of American Indian (AI) and Asian American, Native Hawaiian and Pacific Islander (AANHPI) racial/ethnic groups on death certificates. We conducted probabilistic sensitivity analysis via Monte Carlo simulations, with 10,000 replications, to derive bias-adjusted estimates for the association between race/ethnicity and COVID-19 mortality (in a case–control approach) along with the 2.5th and 97.5th simulation limits [35, 36]. In this analysis, COVID-19 decedents whose race/ethnicity was derived from death certificates were considered “cases” while CA worker population estimates whose self-identified race/ethnicity values were from CPS were considered “controls”. Those identified as AI and AANHPI (in separate analyses) represented “exposed” while the reference racial/ethnic group White represented “unexposed”. We analyzed a scenario of misclassification of exposure (race/ethnicity) differential by outcome (that only applied to decedents) that would mainly be generated by under ascertainment of the exposed racial groups in death certificates [33].

Since self-identified race/ethnicity from CPS estimates (“controls”) are considered as standard for race/ethnicity determination [33], and given the high accuracy of reports of race/ethnicity for white populations in death certificates (“cases unexposed”) [33], we assumed sensitivity and specificity of 0.99 to 1 for CPS estimates of AI and AANHPI ( “controls exposed”) and white (“controls unexposed”) respectively, and specificity of 0.99 to 1 for death certificate reports of white (“cases unexposed”). Based on previous underreporting of AI in death certificates, we assumed a sensitivity of 0.55 to 0.85 (“cases exposed”) [33, 34]. We assumed a sensitivity of 0.85 to 0.95 for AANHPI identification in death certificates [33]. All bias parameters used uniform distribution.

Considering potential inaccuracies in reporting of education level, we conducted another sensitivity analysis combining master’s, doctorate, and professional degree holders into one graduate degree category.

Finally, since our study was restricted to the workforce (i.e., decedents likely or confirmed to be working prior to death) and since both demographic information and predictors of mortality, such as health status and comorbidities, could affect employment status, we conducted a third sensitivity analysis to examine these associations in the entire California population aged 18–64 years without restriction by work status. SAS 9.4 (SAS Institute, Cary, NC) was used for generating the analytic dataset and for data analysis.

## Results

The characteristics of the worker study population are presented in Table 1. Overall, of the 38,872,799 Californians and 48,325 COVID-19 deaths, 24,133,578 workers and 26,068 COVID-19 decedents were included in the study. Among COVID-19 decedents, there was overrepresentation of those aged 50–64 (72.2% of COVID-19 decedents vs 29.7% of the population), Latinos (61% vs 40% of the population), those with the lowest education levels (66.2% vs 39.9% of the population) and foreign-born workers (51.0% vs 32.7% of the population). Foreign-born status, education level and race/ethnicity were unknown for 4.1%, 3.5% and < 1%, respectively, of the COVID-19 decedents. There were no missing values for other characteristics reported.

**Table 1:** Characteristics of California worker COVID-19 decedents and working age population, aged 18–64 years, January 2020 to May 2022

Characteristics	COVID-19 decedents (*n* = 26,068)		CA worker population (*n* = 24,133,578)	
**Age**				
18–29	679	2.6%	6,391,077	26.5%
30–39	1,998	7.7%	5,656,737	23.4%
40–49	4,581	17.6%	4,909,753	20.3%
50–59	10,268	39.4%	4,860,748	20.1%
60–64	8,542	32.8%	2,315,263	9.6%
**Sex**				
Female	8,406	32.2%	12,109,222	50.2%
Male	17,662	67.8%	12,024,356	49.8%
**Race/ethnicity**				
White	5,263	20.2%	9,034,853	37.4%
African American	2,113	8.1%	1,419,552	5.9%
Latino	15,900	61.0%	9,627,600	39.9%
Asian American	1,883	7.2%	3,769,006	15.6%
American Indian	209	0.8%	117,661	0.5%
Native Hawaiian and Other Pacific Islander	284	1.1%	144,600	0.6%
Multi-Race & other	416	1.6%	20,306	0.1%
**Foreign-born**				
US-born	12,780	49.0%	16,234,534	67.3%
Foreign-born	13,288	51.0%	7,899,044	32.7%
**Education**				
GED, HS Graduate or less	17,253	66.2%	8,911,113	36.9%
Bachelor’s & associate degree including some College	7,186	27.6%	12,225,648	50.7%
Master's	420	1.6%	2,261,144	9.4%
Professional	185	0.7%	287,945	1.2%
Doctorate	104	0.4%	447,728	1.9%
**Marital Status**				
Not married	13,105	50.3%	12,674,310	52.5%
Married	12,963	49.7%	11,459,268	47.5%

Professional includes MD, DDS, DVM, LLB, and JD

The crude mortality rate (MR) according to six demographic characteristics for each wave among workers is shown in Table 2. COVID-19 mortality among workers was highest during Wave 3 (*n* = 8759, MR per 100,000 = 50.40), followed by Wave 4 (*n* = 4975, MR per 100,000 = 27.92) (Table 2). In all waves, males had higher COVID-19 MR than females; COVID-19 MR increased as age group increased; those with the lowest education level consistently had the highest mortality rates; foreign-born had higher rate of COVID-19 mortality than US-born and those who were married had slightly higher unadjusted mortality rates than those not married. By race/ethnicity, Native Hawaiian and Other Pacific Islanders had the highest mortality rate, followed by Latinos, during Waves 2 and 3. Whites had the lowest mortality rate in Waves 1, 2 and 3, while Asians had the lowest rate in later waves (Waves 4 and 5).

**Table 2:** Crude COVID-19 mortality rate per 100,000 by worker characteristics

Characteristics	Wave 1 (Mar 2020-Jun 2020)	Wave 2 (Jul 2020-Nov 2020)	Wave 3/ Epsilon & Alpha variants (Dec 2020-May 2021)	Wave 4/Delta (Jun 2021-Jan 2022)	Wave 5/Omicron (Feb 2022-May 2022)
**All Workers**	9.51	19.26	50.40	27.92	15.24
**Age**					
18–29	0.78	1.61	2.87	2.82	1.37
30–39	2.68	4.49	10.27	10.03	3.75
40–49	6.52	13.35	31.89	24.61	10.57
50–59	15.81	33.72	86.49	47.46	25.11
60–64	35.58	74.06	201.84	87.11	57.69
**Sex**					
Female	4.07	9.09	24.17	15.16	8.51
Male	11.44	23.59	60.31	33.47	17.60
**Race/ethnicity**					
White	2.49	5.64	17.26	19.44	11.29
African American	9.40	20.42	51.27	43.12	23.25
Latino	15.25	30.64	72.46	31.73	15.91
Asian American	4.41	8.07	25.68	10.31	5.34
American Indian	6.40	16.11	61.99	74.69	33.11
Native Hawaiian and Other Pacific Islander	12.28	62.14	82.79	54.43	20.29
**Foreign-born**					
US-born	4.12	9.98	26.67	24.64	12.72
Foreign-born	16.68	31.53	80.25	25.90	14.72
**Education**					
GED, HS Graduate or less	18.02	36.61	83.88	45.58	23.44
Bachelor’s & associate degree including some College	3.72	8.40	25.34	17.44	9.22
Master's	1.55	2.89	6.36	5.22	3.26
Professional	4.14	9.80	25.65	15.22	8.06
Doctorate	1.95	3.40	10.98	3.75	3.08
**Marital Status**					
Not married	7.85	16.15	39.06	22.66	13.03
Married	8.37	17.74	48.47	27.76	13.81

Mortality count in each Wave was Wave 1: 1665, Wave 2:3378, Wave 3: 8759, Wave 4: 4975, Wave 5: 2754

**Fig. 1:**
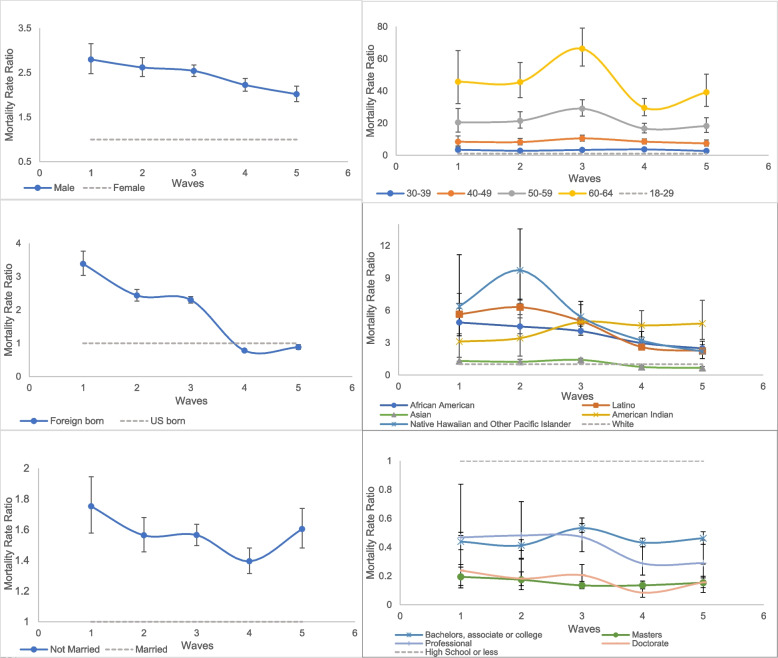
Adjusted mortality rate ratios for **a**) male compared to female, **b**) each Age group compared to 18–29, **c**) Foreign-born compared to US-born, **d**) each race/ethnicity compared to White, **e**) not Married compared to married and **f**) each education level compared to those with education of high school graduate or less. Note: Wave 1: Mar 2020-Jun 2020, Wave 2: Jul 2020-Nov 2020, Wave 3/Epsilon & Alpha: Dec 2020-May 2021, Wave 4/Delta: Jun 2021-Jan 2022, Wave 5/Omicron: Feb 2022-May 2022. Significant changes in disparity over wave for each characteristic based on the test of interaction at an alpha level of 0.05 were as follows: 1a) by sex: Wave 3 to 4 (decline in disparity); 1b) by age group: Wave 2 to 3-groups 50–59 and 60–64 (increase); Wave 3 to 4-groups 50–59 and 60–64 (decline); 1c) by foreign-born status: Wave 1 to 2 and Wave 3 to 4 (decline); and Wave 4 to 5 (increase); 1d) by race/ethnicity: Wave 2 to 3- Latino and Native Hawaiian (decline); Wave 3 to 4- African American, Latino, Asian and Native Hawaiian (decline); Wave 4 to 5- African American (decline); and 1e) by marital status: Wave 4 to 5 (disparity increase); and 1f) by education level: Wave 2 to 3 and Wave 3 to 4- Bachelor’s degree (decline in disparity)

According to the adjusted models shown in Table 3, compared to females, male workers had a greater mortality rate in all waves. The differences by sex narrowed from Waves 1 to 5, with the highest disparity observed in Wave 1 (MRR = 2.80, 95% CI (2.48 to 3.15)), the smallest observed in Wave 5 (MRR = 2.02, 95% CI (1.85 to 2.20)) and the largest change observed between Waves 3 and 4 (Fig. 1a).

**Table 3:** Adjusted Mortality Rate Ratio (MRR) with 95%CI for working California population comparing each characteristic to their respective referent groups

	**Wave 1**	**Wave 2**	**Wave 3/ Epsilon & Alpha variants**	**Wave 4/Delta**	**Wave 5/Omicron**
**Characteristics**	**(Mar 2020-Jun 2020)**	**(Jul 2020-Nov 2020)**	**(Dec 2020-May 2021)**	**(Jun 2021-Jan 2022)**	**(Feb 2022-May 2022)**
**Age (ref-18–29)**					
30–39	3.43 (2.35 to 5.03) (0.02)	2.80 (2.14 to 3.65) (0.02)	3.34 (2.75 to 4.05) (0.02)	3.66 (3.02 to 4.44) (0.02)	2.67 (2.01 to 3.55) (0.02)
40–49	8.41 (5.86 to 12.06) (0.02)	8.21 (6.43 to 10.47) (0.02)	10.5 (8.76 to 12.59) (0.02)	8.48 (7.06 to 10.19) (0.02)	7.39 (5.69 to 9.6) (0.02)
50–59	20.46 (14.42 to 29.03) (0.02)	21.42 (16.93 to 27.11) (0.02)	28.99 (24.3 to 34.58) (0.02)	16.61 (13.88 to 19.86) (0.02)	18.18 (14.12 to 23.41) (0.02)
60–64	45.75 (32.14 to 65.12) (0.02)	45.53 (35.92 to 57.72) (0.02)	66.29 (55.53 to 79.13) (0.02)	29.53 (24.62 to 35.42) (0.02)	39.18 (30.4 to 50.5) (0.02)
**Sex (ref-female)**					
Male	2.80 (2.48 to 3.15) (0.01)	2.62 (2.41 to 2.84) (0.01)	2.54 (2.41 to 2.67) (0.01)	2.22 (2.09 to 2.37) (0.01)	2.02 (1.85 to 2.2) (0.01)
**Race/ethnicity (ref-white)**					
African American	4.88 (3.84 to 6.21) (0.02)	4.51 (3.84 to 5.31) (0.02)	4.09 (3.68 to 4.53) (0.02)	2.97 (2.68 to 3.3) (0.02)	2.48 (2.15 to 2.85) (0.02)
Latino	5.65 (4.8 to 6.66) (0.02)	6.30 (5.62 to 7.06) (0.02)	5.02 (4.69 to 5.37) (0.02)	2.61 (2.42 to 2.81) (0.02)	2.25 (2.03 to 2.48) (0.02)
Asian American	1.31 (1.03 to 1.66) (0.04)	1.24 (1.05 to 1.47) (0.04)	1.41 (1.28 to 1.56) (0.02)	0.76 (0.67 to 0.87) (0.02)	0.67 (0.56 to 0.8) (0.02)
American Indian	3.11 (1.28 to 7.56) (0.04)	3.43 (1.77 to 6.64) (0.04)	4.93 (3.71 to 6.54) (0.02)	4.62 (3.55 to 5.99) (0.02)	4.78 (3.3 to 6.93) (0.02)
Native Hawaiian and Other Pacific Islander	6.39 (3.65 to 11.17) (0.02)	9.72 (6.96 to 13.57) (0.02)	5.41 (4.28 to 6.84) (0.02)	3.22 (2.56 to 4.04) (0.02)	2.19 (1.53 to 3.15) (0.02)
**Foreign born (ref-US-born)**					
Foreign born	3.38 (3.04 to 3.76) (0.01)	2.43 (2.27 to 2.61) (0.01)	2.30 (2.2 to 2.4) (0.01)	0.78 (0.73 to 0.83) (0.01)	0.88 (0.81 to 0.95) (0.01)
**Education (ref-HS)**					
Bachelors, associate, or College	0.44 (0.38 to 0.5) (0.01)	0.41 (0.38 to 0.45) (0.01)	0.53 (0.5 to 0.56) (0.01)	0.43 (0.4 to 0.46) (0.01)	0.46 (0.42 to 0.51) (0.01)
Masters	0.19 (0.13 to 0.28) (0.01)	0.17 (0.13 to 0.23) (0.01)	0.13 (0.11 to 0.16) (0.01)	0.14 (0.11 to 0.16) (0.01)	0.15 (0.12 to 0.2) (0.01)
Professional	0.47 (0.26 to 0.84) (0.06)	0.48 (0.32 to 0.72) (0.06)	0.47 (0.37 to 0.6) (0.06)	0.29 (0.21 to 0.4) (0.06)	0.29 (0.19 to 0.45) (0.06)
Doctorate	0.24 (0.12 to 0.48) (0.01)	0.18 (0.1 to 0.32) (0.01)	0.21 (0.15 to 0.28) (0.01)	0.08 (0.05 to 0.14) (0.01)	0.16 (0.09 to 0.28) (0.01)
**Marital Status**					
Not married	1.75 (1.58 to 1.95) (0.01)	1.56 (1.46 to 1.68) (0.01)	1.57 (1.50 to 1.64) (0.01)	1.39 (1.31 to 1.48) (0.01)	1.60 (1.48 to 1.74) (0.01)

Estimates were derived from separate models for each characteristic in each wave. Romano–Wolf adjusted p-values are shown in parentheses next to 95% Cis

Compared to those aged 18–29 years, each age group increase among workers was associated with an increase in mortality in all waves. The differences in MRR according to age were greatest in Wave 3 and smallest in Wave 4. Among those aged 50–59 and 60–64 years, there was an increase in the MRR from Waves 2 to 3 and a decrease in the MRR from Waves 3 to 4. Overall, disparities by age did not change substantially across waves (Fig. 1b).

Compared to US-born workers, foreign-born workers had a greater mortality rate in most waves except in Waves 4 and 5 (during the Delta and Omicron variants). The disparity between foreign-born and US-born workers substantially decreased from Wave 1 (MRR = 3.38, 95% CI (3.04 to 3.76)) to Wave 5 (MRR = 0.88 95% CI (0.81 to 0.95)) (Fig. 1c).

By race/ethnicity, Native Hawaiian, Latino, and African American workers had higher mortality rates than Whites in all waves. We observed substantial changes in racial disparities for the following transitions: Waves 2 to 3 for declines in MRR among Latino and Native Hawaiian Americans; Waves 3 to 4 for declines in MRR among African Americans; Latino, Asian, and Native Hawaiian; and Waves 4 to 5 for reductions in MRR among African Americans. Overall, in Waves 4 and 5 (during delta and omicron variants), disparities drastically decreased for most racial groups (Native Hawaiian, Latino, African American and Asian), except for Native Americans (Fig. 1d). In sensitivity bias analysis, the bias corrected estimates for AI (OR wave 1 to 5 = 5.3, 95%CI (3.2, 20.4) to 6.2, 95%CI (3.9, 13.4)) were larger than the observed estimates (OR wave 1 to 5 = 2.4 (1.0, 5.9) to 3.0 (2.1, 4.3)) but showed a similar pattern in which the AI racial group continued to have higher relative mortality than White in later waves, particularly during Delta variant. The bias corrected estimates for the combined AANHPI group showed larger estimates than observed but declining relative mortality in later waves similar to the pattern for Asian Americans. The observed estimates for AI were biased towards the null whereas those for AANHPI were biased toward the null for waves 1,2 and 3 and away from the null in waves 4 and 5 (Table 4).

**Table 4:** Probabilistic sensitivity analysis for misclassification of race/ethnicity in death certificates for the association between race/ethnicity and COVID-19 mortality among working California population. Odds Ratio (OR) with 95% confidence limits

American Indian	Wave 1 (Mar 2020-Jun 2020)	Wave 2 (Jul 2020-Nov 2020)	Wave 3/ epsilon & alpha variants (Dec 2020-May 2021)	Wave 4/Delta (Jun 2021-Jan 2022)	Wave 5/omicron (Feb 2022-May 2022)
Observed OR	2.4 (1.0 to 5.9)	2.7 (1.4 to 5.3)	3.5 (2.6 to 4.6)	3.9 (3.0 to 5.0)	3.0 (2.1 to 4.3)
Bias corrected OR	5.3 (3.2 to 20.4)	6.4 (3.6 to 94.7)	7.7 (4.6 to 22.1)	9.3 (5.2 to 42.6)	6.2 (3.9 to 13.4)
**Asian, Native Hawaiian and Pacific Islander**					
Observed OR	1.9 (1.5 to 2.3)	1.6 (1.4 to 1.9)	1.6 (1.4 to 1.7)	0.63 (0.56 to 0.7)	0.55 (0.47 to 0.64)
Bias corrected OR	2.3 (2.1 to 2.5)	1.9 (1.8 to 2.1)	1.9 (1.7 to 2.0)	0.71 (0.67 to 0.76)	0.62 (0.58 to 0.66)

Estimates of MRR from bivariate analyses with Poisson regression produced the same results as the observed ORs

By marital status, those who were not married had higher adjusted MRRs in all waves than did those who were married. Disparity by marital status narrowed from Wave 1 to Waves 3 and 4 (MRR in Wave 4 = 1.39, 95% CI (1.31 to 1.48)) but increased again in Wave 5 (MRR = 1.60, 95% CI (1.48 to 1.74)), with an increased risk for those who were not married (Fig. 1e).

According to education level, those with a graduate degree had a lower MRR than did those with other education levels in most waves (2, 3 and 4), while those with a high school education or less (ref group) had the highest mortality rate in all waves (Fig. 1f). The mortality rate gap between those with an education level of high school or less and those with a bachelor’s degree narrowed from Waves 2 (July 2020-November 2020) to 3 (December 2020-May 2021) and widened from Waves 3 to 4. In Waves 4 and 5 (during the Delta and Omicron variants), the difference in mortality rate between individuals with lower education levels and those with professional and doctorate degrees increased further (Table 3, Fig. 1f). Generally, changes in disparities by education level were modest across all waves. In sensitivity analysis combining graduate degrees, the education gradient was stronger in which those Bachelor’s, associate or college degree had consistently lower MRR than those with education level of HS or less while those with graduate degrees had even lower relative mortality rates throughout the pandemic. This gap was most pronounced during Delta variant (Table 5).

**Table 5:** Sensitivity analysis combining Masters, Doctorate, and Professional degrees for the association between education level and COVID-19 mortality among working California population. Adjusted Mortality Rate Ratio (MRR) with 95%CIs

Education (ref-HS)	Wave 1 (Mar 2020-Jun 2020)	Wave 2 (Jul 2020-Nov 2020)	Wave 3/ epsilon & alpha variants (Dec 2020-May 2021)	Wave 4/delta (Jun 2021-Jan 2022)	Wave 5/omicron (Feb 2022-May 2022)
Bachelors, associate, or College	0.43 (0.38 to 0.5)	0.41 (0.37 to 0.45)	0.53 (0.5 to 0.56)	0.43 (0.4 to 0.46)	0.46 (0.42 to 0.51)
Graduate Degree	0.23 (0.17 to 0.31)	0.21 (0.17 to 0.26)	0.19 (0.16 to 0.21)	0.14 (0.12 to 0.17)	0.17 (0.14 to 0.21)

According to our sensitivity analysis excluding the restriction of work status, we found that the magnitudes and trends of the associations between each characteristic and COVID-19 mortality were similar but were slightly attenuated (supplementary Table 1).

## Discussion

According to our temporal analysis of workers who died of COVID-19 in California, male sex; being aged 50–64 years; being Native Hawaiian or Latino or African American race/ethnicity; having an education level of high school or less; and not being married were associated with a higher COVID-19 mortality rate in all waves. While disparities by sex, race/ethnicity and foreign-born status narrowed in waves 4 and 5 (post widespread vaccine availability), with the largest decline occurring from Wave 3 to 4, there were minimal changes in disparities by age, education level and marital status across waves.

Sex disparities in COVID-19 incidence and mortality among men have been attributed to underlying social and contextual factors such as preexisting health status, health behaviors, occupation and social experience [3]. Once tested, men were more likely to be hospitalized and to suffer severe outcomes [37], possibly due to underlying health conditions such as cardiovascular disease, which are more common in men [38, 39]. One study found that despite similar proportions of males and females with confirmed COVID-19, male patients had more severe health outcomes, more hospitalizations and more deaths than female patients [40]. However, vaccination likely prevented adverse outcomes and may have narrowed the gap in COVID-19 mortality rates between men and women in later waves of the pandemic. Societal and cultural differences in the implementation of mitigation measures over time may also play a role in the change in disparities by sex. Earlier in the pandemic, women reported taking more precautions than men, such as cancelling travel and large gatherings, stocking food and household supplies and staying home to reduce their exposure [41]. Women also left the workforce in greater numbers than men during the pandemic to care for children or family members in need [42]. Differences in the implementation of preventive behaviors by sex as well as the associated impact of these measures on sex disparities may have decreased in the periods where restrictions were lifted and vaccinations were available, providing protection for both male and female workers.

Racial disparities were apparent early in the pandemic and may be attributed to greater structural inequities, a greater prevalence of comorbidities and the impact of social determinants of health, such as overrepresentation of people of color in low-wage jobs [11, 14, 43]. Similar to our findings, other studies have shown that racial disparities declined in later stages of the pandemic [44, 45], which could be due to increasing vaccine uptake among racial groups over time. One study showed that COVID-19 vaccine hesitancy decreased by one-third from January to May 2021 (waves 3 to 4), with relatively large decreases in hesitancy among Black, Pacific Islander and Hispanic participants [45]. In our analysis, these racial groups had the highest MRRs compared to Whites in earlier waves; a substantial increase in vaccine uptake among these groups could explain the declining disparities by race/ethnicity we observed in later waves of the pandemic. The findings from the bias analysis for the AANHPI group support this decline in relative rates in post-vaccine availability waves. The bias-corrected estimates for AI show that the disproportionate burden of COVID-19 deaths among AI relative to the white population could be even higher in the absence of misclassification. Despite declines for other racial groups, Native Americans continued to be disproportionately affected by COVID-19 mortality in the post-vaccine availability waves.

Disparities by race/ethnicity and foreign-born status may be closely related, as a majority of the foreign-born workers (57.7%) were Latino [46]. This may therefore partly explain the similar pattern of narrowing in disparities by race/ethnicity and by foreign-born status. Previous studies also showed that immigrants were disproportionately affected by COVID-19, particularly earlier during the pandemic [47, 48]. One study found that the majority of US-born mortalities were among nonworking residents of long-term care facilities and occurred late in 2020, while foreign-born mortalities occurred outside of residential institutions and earlier during the pandemic [47]. Our results also showed that, compared to US-born workers, foreign-born workers were more than three times more likely to die from COVID-19 earlier during the pandemic (in Wave 1) and 12% less likely to die in Wave 5. Immigrants were overrepresented in multiple sectors that were frontline and most affected by the pandemic [48, 49]. Less stable employment conditions, limited teleworking possibilities and obstacles to health services, such as lack of accurate information and language barriers, were among the factors that contributed to the disproportionate impact on foreign-born individuals at the beginning of the pandemic [48, 49]. Improvements in COVID-19 prevention and care later in the pandemic (including better information, increased testing, vaccinations and interventions) may have helped reduce disparities caused by immigration status, as the evidence for long-term disparities is mixed [48]. Data from Urban Institute’s Well-Being and Basic Needs Survey shows that immigrant families in CA had higher perception of exposure to COVID-19, were more likely to report intent to get vaccinated and more likely to trust state or local public health officials than non-immigrant families [50]. This may have contributed to the lower mortality rates seen among immigrants in later waves.

Like previous studies, we found that older individuals (50 to 64 years) were more likely to die from COVID-19 than younger individuals [2, 4], likely because a greater incidence of comorbidities and weakened immune systems contribute to more severe outcomes [4]. Compared to that in individuals aged 18–29 years, the high mortality rate in Wave 3 for those aged 50 to 64 years may be due to the overall increase in infection during this period. As infection rates rose from the end of 2020 through early 2021, the older population had higher mortality rates than younger individuals [2], therefore increasing the disparity between older and younger individuals. The large decline in the MRR for older individuals between Waves 3 (Epsilon & Alpha variants) and 4 (delta variant) may be due to the vaccinations introduced during this period. Since older individuals were prioritized for receiving COVID-19 vaccinations earlier than the general population [51], early protection would lower mortality rates among older worker populations and overall relative disparity compared to younger individuals.

Our findings showed that individuals with an education level of high school or less had consistently greater COVID-19 mortality rates throughout the pandemic, similar to the findings of another study in which excess COVID-19 mortality decreased as educational level increased [5]. The high mortality rate among those with less education could be due to upstream socioeconomic factors that create few work opportunities and lead to lower income levels and precarious social status, all of which can in turn affect SARS-CoV-2 exposure, limit access to healthcare and increase the risk of comorbidities [5]. Persons working in low-wage jobs have less ability to telework, increasing the chance for workplace exposure but fewer opportunities for paid sick leave [52]. The disparities by education may also be mediated by occupation and industry, as education is a strong predictor of occupation and type of occupation is associated with COVID-19 mortality [12, 14]. A substantial proportion of frontline workers, such as those in Production, Transportation and Farming occupations, comprised less educated and disadvantaged minority workers [53] who suffered excess COVID-19 mortality during the pandemic [14]. Disparities by education level slightly declined during the largest peak in mortality (Wave 3) and increased in the wide vaccination era (Wave 4). The disproportionate mortality among less educated groups may have been influenced by vaccine hesitancy and access and lower vaccination levels perpetuated through later waves [54]. Disparities by education level and occupation have persisted during the pandemic despite the availability of COVID-19 vaccines [5, 14], as shown by trends that differed from those observed for race/ethnicity, sex and foreign-born status, which showed continuous narrowing in later waves. Findings from sensitivity analysis combining graduate degrees also showed the continuation of this disparity in the post-vaccine availability waves. The gaps in COVID-19 mortality rate by education level were even more apparent and death rates substantially lowered with increasing educational attainment.

Throughout all waves, we found that individuals who were not married had higher mortality rates than those who were married. Previous studies have shown that being unmarried is associated with adverse COVID-19 outcomes compared to being married [55, 56], which parallels the overall higher death rate for unmarried persons, particularly men [57].

We documented socioeconomic inequalities in COVID-19 mortality, which, as shown by previous studies [58–61], mirrored, and possibly exacerbated preexisting differentials. The disproportionate COVID-19 mortality among certain populations may reflect factors that increase exposure to COVID-19, such as overrepresentation in low-wage jobs and the essential workforce; inadequate safety and mitigation policies in the workplace; and differential vulnerability to severe outcomes resulting from underlying health conditions, comorbidities, and socioeconomic status. Prevention policies that address different levels of these gaps could help narrow disparities by worker characteristics. These trends in disparities suggest that policies and interventions may have helped buffer against some of these disparities later in the pandemic but may not have been enough to eliminate them and may therefore indicate remaining gaps. Further studies could be useful for evaluating the role of specific interventions in reducing disparities in COVID-19 outcomes.

Limitations of this study include the potential presence of residual confounding, the potential for misclassification and a lack of data on key factors that may have impacted the trends in worker disparities. First, the path from worker characteristics to SARS-CoV-2 exposure to COVID-19 mortality is complex and may be affected, mediated, modified or confounded by different biological, individual, societal or environmental factors [62]. Therefore, residual confounding likely occurred even though we adjusted for covariates identified as confounders. Second, there could be misclassification of demographic characteristics as well as outcomes, particularly earlier during the pandemic, when undiagnosed COVID-19 fatalities may have been reported as non-COVID-19 deaths [63]. Inequities in testing earlier during the pandemic may have contributed to differential diagnoses among subgroups of the population [64], which may have led to underreporting of COVID-19 mortality and decreased accuracy among certain groups early in the pandemic. However, given that our data include all COVID-19 mortality among the worker population in California during the specified period, we expect the effect of differential underreporting on our estimates to be small. Additionally, use of two different data sources and inaccuracies in reporting in death certificates could induce misclassification bias. However, we do not expect these inaccuracies to be largely systematic except for reports on high school graduation [65], which would not affect our estimates based on our categorization of educational level. Therefore, like previous studies that used similar data sources to study disparities [5, 22], we expect other differences in reporting to have minimal effects on the patterns we observed. Finally, we did not include data on workers’ vaccination status, which may have had a substantial impact on the patterns of disparities observed later in the pandemic. Since we included all California workers and COVID-19 decedents eligible for inclusion, our study is generalizable to California and states with similar worker populations.

## Conclusion

Our findings demonstrate disparities in COVID-19 mortality according to worker characteristics in all waves of the pandemic. Workers aged 50–64 years, male, Native Hawaiian, Latino, African American, foreign-born, with a high school education or less and not married were disproportionately affected by COVID-19 mortality. While disparities by sex, race/ethnicity and foreign-born status narrowed in later waves/post vaccine availability, particularly during the Delta and Omicron variants, there were minimal changes in disparities by age, education level and marital status across waves. Disparities in COVID-19 mortality are a reflection and amplification of preexisting health inequities [58, 61]. Tailoring interventions to high-risk populations and addressing underlying social and structural issues are critical for achieving health equity.

### Supplementary Information

Supplementary Material 1.

## Data Availability

Descriptive Metadata is included in supplementary files.Per the Center for Health Statistics and Informatics of the California Department of Public Health, data cannot be shared publicly because they contain personal and confidential information. Requests for data should be communicated to the Center for Health Statistics and Informatics at vog.ac.hpdc@srih.

## References

[1] COVID-19 Was Third Leading Cause of Death in U.S. April 2022. US DEPARTMENT OF HEALTH AND HUMAN SERVICES Centers for Disease Control and Prevention https://www.cdcgov/media/releases/2022/s0422-third-leading-causehtml. Accessed December 8, 2022.;

[2] Mehraeen E, Karimi A, Barzegary A (2020). Predictors of mortality in patients with COVID-19-a systematic review. Eur J Integr Med.

[3] Danielsen AC, Lee KM, Boulicault M (2022). Sex disparities in COVID-19 outcomes in the United States: Quantifying and contextualizing variation. Soc Sci Med.

[4] Yanez ND, Weiss NS, Romand JA, Treggiari MM. COVID-19 mortality risk for older men and women. BMC Public Health. Nov 19 2020;20(1):1742. 10.1186/s12889-020-09826-8 10.1186/s12889-020-09826-8 PMC7675386 33213391

[5] Chen YH, Matthay EC, Chen R, et al. Excess Mortality in California by Education During the COVID-19 Pandemic. Am J Prev Med. Jul 27 2022;10.1016/j.amepre.2022.06.020 10.1016/j.amepre.2022.06.020 PMC9325680 36114132

[6] Feldman JM, Bassett MT (2021). Variation in COVID-19 Mortality in the US by Race and Ethnicity and Educational Attainment. JAMA Netw Open.

[7] Case A, and Angus Deaton. MORTALITY RATES BY COLLEGE DEGREE BEFORE AND DURING COVID-19. Working Paper Working Paper Series National Bureau of Economic Research https://doi.org/103386/w29328. Oct 2021;

[8] Paglino E, Elo IT (2024). Immigrant mortality advantage in the United States during the first year of the COVID-19 pandemic. Demogr Res Jan-Jun.

[9] Garcia E, Eckel SP, Chen Z, Li K, Gilliland FD (2021). COVID-19 mortality in California based on death certificates: disproportionate impacts across racial/ethnic groups and nativity. Ann Epidemiol.

[10] Riley AR, Chen YH, Matthay EC (2021). Excess mortality among Latino people in California during the COVID-19 pandemic. SSM Popul Health.

[11] Mude W, Oguoma VM, Nyanhanda T, Mwanri L, Njue C. Racial disparities in COVID-19 pandemic cases, hospitalisations, and deaths: A systematic review and meta-analysis. J Glob Health. Jun 26 2021;11:05015. 10.7189/jogh.11.05015 10.7189/jogh.11.05015 PMC8248751 34221360

[12] Cummings KJ, Beckman J, Frederick M (2022). Disparities in COVID-19 fatalities among working Californians. PLoS ONE.

[13] Goldman N, Pebley AR, Lee K, Andrasfay T, Pratt B. Racial and ethnic differentials in COVID-19-related job exposures by occupational standing in the US. medRxiv. Apr 6 2021;10.1101/2020.11.13.20231431 10.1371/journal.pone.0256085 PMC8409606 34469440

[14] Chen YH, Riley AR, Duchowny KA (2022). COVID-19 mortality and excess mortality among working-age residents in California, USA, by occupational sector: a longitudinal cohort analysis of mortality surveillance data. Lancet Public Health.

[15] Paul LJ (2023). COVID-19 May Have Been Job Related for One Fourth of Diagnosed Adults. Am J Public Health.

[16] Gaffney A, Himmelstein DU, McCormick D, Woolhandler S (2023). COVID-19 Risk by Workers' Occupation and Industry in the United States, 2020–2021. Am J Public Health.

[17] Short SE, Mollborn S (2015). Social Determinants and Health Behaviors: Conceptual Frames and Empirical Advances. Curr Opin Psychol.

[18] Social Determinants of Health: Know What Affects Health. March 2021. Centers for Disease Control and Preventionhttps://www.cdc.gov/socialdeterminants/abouthtml. Accessed December 8, 2022. ;

[19] Dalsania AK, Fastiggi MJ, Kahlam A (2022). The Relationship Between Social Determinants of Health and Racial Disparities in COVID-19 Mortality. J Racial Ethn Health Disparities.

[20] Braveman P, Gottlieb L. The social determinants of health: it's time to consider the causes of the causes. Public Health Rep. Jan-Feb 2014;129 Suppl 2(Suppl 2):19–31. 10.1177/00333549141291s206 10.1177/00333549141291S206 PMC3863696 24385661

[21] COVID-19 Prevention Actions. October 2022. Centers for Disease Control and Prevention: COVID-19 https://www.cdc.gov/coronavirus/2019-ncov/prevent-getting-sick/preventionhtml. Accessed December 10, 2022. ;

[22] Matthay EC, Duchowny KA, Riley AR, et al. Occupation and Educational Attainment Characteristics Associated With COVID-19 Mortality by Race and Ethnicity in California. JAMA Netw Open. Apr 1 2022;5(4):e228406. 10.1001/jamanetworkopen.2022.8406 10.1001/jamanetworkopen.2022.8406 PMC9034406 35452107

[23] Shao Y, Ahmed A, Liappis AP, Faselis C, Nelson SJ, Zeng-Treitler Q (2021). Understanding Demographic Risk Factors for Adverse Outcomes in COVID-19 Patients: Explanation of a Deep Learning Model. J Healthc Inform Res.

[24] How trends have changed in California. The New York Times https://www.nytimes.com/interactive/2023/us/california-covid-caseshtml. Accessed April 10, 2024.;

[25] COVID-19 Time-Series Metrics by County and State (ARCHIVED) - Dataset - California Health and Human Services Open Data Portal. California Department of Public Health. https://data.chhs.ca.gov/dataset/covid-19-time-series-metrics-by-county-and-state. Accessed 15 Apr 2024.

[26] Epling S. Coronavirus Variants: The Delta Variant and the Future Impact of Viral Mutations. CAS A division of the American Chemical Society https://www.cas.org/resources/blog/coronavirus-variants. Jul 2021;

[27] Staff LAT. Tracking COVID-19 in California. Los Angeles Times https://www.latimes.com/projects/california-coronavirus-cases-tracking-outbreak/. Dec 2023;

[28] Rashedi R, Samieefar N, Masoumi N, Mohseni S, Rezaei N (2022). COVID-19 vaccines mix-and-match: The concept, the efficacy and the doubts. J Med Virol.

[29] Gebreegziabher E, Bui D, Cummings KJ, et al. Temporal assessment of disparities in California COVID-19 mortality by industry: a population-based retrospective cohort study. Annals of Epidemiology. 2023/11/01/ 2023;87:51–59.e2. 10.1016/j.annepidem.2023.09.003 10.1016/j.annepidem.2023.09.003 37714416

[30] Sarah Flood  MK, Renae Rodgers, Steven Ruggles, Robert Warren J , Michael Westberry (2021). Integrated Public Use Microdata Series, Current Population Survey: Version 9.0.

[31] Leppink J, O'Sullivan P, Winston K (2017). Are differences between groups different at different occasions?. Perspect Med Educ.

[32] Clarke D, Romano JP, Wolf M. The Romano–Wolf multiple-hypothesis correction in Stata. The Stata Journal. 2020/12/01 2020;20(4):812–843. 10.1177/1536867X20976314

[33] Arias E, Heron M; National Center for Health Statistics; Hakes J; US Census Bureau. The Validity of Race and Hispanic-origin Reporting on Death Certificates in the United States: An Update. Vital Health Stat 2. 2016;(172):1-21. PMID:28436642. 28436642

[34] Jim MA, Arias E, Seneca DS, et al. Racial misclassification of American Indians and Alaska Natives by Indian Health Service Contract Health Service Delivery Area. Am J Public Health. Jun 2014;104 Suppl 3(Suppl 3):S295–302. 10.2105/ajph.2014.301933 10.2105/AJPH.2014.301933 PMC4035863 24754617

[35] Lash TL, Fox MP, Fink AK (2011). Applying Quantitative Bias Analysis to Epidemiologic Data.

[36] Orsini N, Bellocco R, Bottai M, Wolk A, Greenland S (2008). A Tool for Deterministic and Probabilistic Sensitivity Analysis of Epidemiologic Studies. Stand Genomic Sci.

[37] Scully EP, Schumock G, Fu M, et al. Sex and gender differences in COVID testing, hospital admission, presentation, and drivers of severe outcomes in the DC/Maryland region. medRxiv. Apr 7 2021;10.1101/2021.04.05.21253827 10.1093/ofid/ofab448 PMC8465334 34584899

[38] Mosca L, Barrett-Connor E, Wenger NK (2011). Sex/gender differences in cardiovascular disease prevention: what a difference a decade makes. Circulation.

[39] Bots SH, Peters SAE, Woodward M. Sex differences in coronary heart disease and stroke mortality: a global assessment of the effect of ageing between 1980 and 2010. BMJ Global Health. 2017;2:e000298. 10.1136/bmjgh-2017-000298 PMC5435266 28589033

[40] Peckham H, de Gruijter NM, Raine C, et al. Male sex identified by global COVID-19 meta-analysis as a risk factor for death and ITU admission. Nat Commun. Dec 9 2020;11(1):6317. 10.1038/s41467-020-19741-6 10.1038/s41467-020-19741-6 PMC7726563 33298944

[41] Kopel J, Perisetti A, Roghani A, Aziz M, Gajendran M, Goyal H. Racial and Gender-Based Differences in COVID-19. Front Public Health. 2020;8:418. 10.3389/fpubh.2020.00418. 10.3389/fpubh.2020.00418 PMC7399042 32850607

[42] Zamarro G, Prados MJ (2021). Gender differences in couples' division of childcare, work and mental health during COVID-19. Rev Econ Househ.

[43] Bui DP, McCaffrey K, Friedrichs M, et al. Racial and Ethnic Disparities Among COVID-19 Cases in Workplace Outbreaks by Industry Sector — Utah, March 6–June 5, 2020. MMWR Morb Mortal Wkly Rep 2020;69:1133–38. 10.15585/mmwr.mm6933e3. 10.15585/mmwr.mm6933e3 PMC7439983 32817604

[44] Riley AR, Kiang MV, Chen Y-H, Bibbins-Domingo K, Glymour MM. Recent Shifts in Racial/Ethnic Disparities in COVID-19 Mortality in the Vaccination Period in California. Journal of General Internal Medicine. 2022/05/01 2022;37(7):1818–1820. 10.1007/s11606-021-07380-6 10.1007/s11606-021-07380-6 PMC8809629 35112284

[45] King WC, Rubinstein M, Reinhart A, Mejia R (2021). Time trends, factors associated with, and reasons for COVID-19 vaccine hesitancy: A massive online survey of US adults from January-May 2021. PLoS ONE.

[46] J WNaB. Frequently Requested Statistics on Immigrants and Immigration in the United States. Migration Policy Institute. 2023;

[47] Horner KM, Wrigley-Field E, Leider JP (2022). A First Look: Disparities in COVID-19 Mortality Among US-Born and Foreign-Born Minnesota Residents. Popul Res Policy Rev.

[48] (COVID-19) OPRtC. What has been the impact of the COVID-19 pandemic on immigrants? An update on recent evidence. 30 August 2022;

[49] Đoàn LN, Chong SK, Misra S, Kwon SC, Yi SS (2021). Immigrant Communities and COVID-19: Strengthening the Public Health Response. Am J Public Health.

[50] Gonzalez D, Michael Karpman, and Hamutal Bernstein. COVID-19 Vaccine Attitudes among Adults in Immigrant Families in California. Urban Institute https://www.urban.org/sites/default/files/publication/103973/covid-19-vaccine-attitudes-among-adults-in-immigrant-families-in-california_0pdf. Apr 2021;

[51] Updated COVID-19 Vaccine Eligibility Guidelines. May 2021. California Department of Public Health. https://www.cdph.ca.gov/Programs/CID/DCDC/Pages/COVID-19/VaccineAllocationGuidelines.aspx. Accessed 8 Dec 2022.

[52] Gallagher S, Roy A, Domeracki SJ (2021). The Low-Wage Essential Worker: Occupational Concerns and Needs in the COVID-19 Pandemic-A Round Table. Workplace Health Saf.

[53] Blau FD, Koebe J, Meyerhofer PA (2021). Who are the essential and frontline workers?. Bus Econ.

[54] Khairat S, Zou B, Adler-Milstein J (2022). Factors and reasons associated with low COVID-19 vaccine uptake among highly hesitant communities in the US. Am J Infect Control.

[55] Harvey R, Hermez M, Schanz L, Karabon P, Wunderlich-Barillas T, Halalau A (2021). Healthcare Disparities Correlated with In-Hospital Mortality in COVID-19 Patients. Int J Gen Med.

[56] Risk of dying from COVID-19 greater for men, unmarried and born in low and middle income countries, Swedish study finds. October 2020. Science News https://www.sciencedaily.com/releases/2020/10/201009102737htm. Accessed December 8, 2022. ;

[57] Curtin SC, Tejada-Vera B. Mortality among adults aged 25 and over by marital status: United States, 2010–2017. NCHS Health E-Stat. 2019. https://www.cdc.gov/nchs/data/hestat/mortality/mortality_marital_status_10_17.htm.

[58] Bambra C, Riordan R, Ford J, Matthews F (2020). The COVID-19 pandemic and health inequalities. J Epidemiol Community Health.

[59] McClure ES, Vasudevan P, Bailey Z, Patel S, Robinson WR (2020). Racial Capitalism Within Public Health-How Occupational Settings Drive COVID-19 Disparities. Am J Epidemiol.

[60] Foster TB, Sonya R. Porter and Nikolas Pharris-Ciurej. “Excess Mortality" During COVID-19 Varied by Race, Ethnicity, Geography (census.gov). United States Census Bureau. Mar 2024. https://content.govdelivery.com/accounts/USCENSUS/bulletins/3902fdf.

[61] Moore JT, Luna-Pinto C, Cox H (2022). Promoting health equity during the COVID-19 pandemic. United States Bull World Health Organ.

[62] Kouser HN, Barnard-Mayers R, Murray E. Complex systems models for causal inference in social epidemiology. J Epidemiol Community Health. Nov 10 2020;10.1136/jech-2019-213052 10.1136/jech-2019-213052 PMC8849440 33172839

[63] Chen Y-H, Stokes AC, Aschmann HE, et al. Excess natural-cause deaths in California by cause and setting: March 2020 through February 2021. PNAS Nexus. 2022;1(3)10.1093/pnasnexus/pgac079 10.1093/pnasnexus/pgac079 PMC9272175 35832865

[64] Kim HN, Lan KF, Nkyekyer E (2020). Assessment of Disparities in COVID-19 Testing and Infection Across Language Groups in Seattle. Washington JAMA Network Open.

[65] Rostron BL, Boies JL, Arias E. Education reporting and classification on death certificates in the United States. Vital Health Stat 2. 2010 May;(151):1-21. PMID: 25093685. 25093685

