# Supplementary material for: Demographic changes in COVID-19 mortality during the pandemic: analysis of trends in disparities among workers using California’s mortality surveillance system
Source: BMC Public Health. 2024 Jul 9;24:1822. doi: 10.1186/s12889-024-19257-4 (PMC11232202; doi:10.1186/s12889-024-19257-4)
Supplement: Supplementary file 1 — Supplementary Material 1. [file 12889_2024_19257_MOESM1_ESM.docx]

| **Supplementary Table 1-** Sensitivity analysis including all California population 18-65: Adjusted Mortality Rate Ratio (MRR) with 95%CI comparing each characteristic to their respective referent groups | | | | | |
| --- | --- | --- | --- | --- | --- |
| **Characteristics** | **Wave 1 (Mar 2020-Jun 2020)** | **Wave 2**  **(Jul 2020-Nov 2020)** | **Wave 3 (Dec 2020-May 2021)** | **Wave 4/Delta**  **(Jun 2021-Jan 2022)** | **Wave 5/Omicron**  **(Feb 2022-May 2022)** |
| **Age (ref-18-29)** |  |  |  |  |  |
| 30-39 | 3.56 (2.61 to 4.87) | 3.25 (2.61 to 4.05) | 3.42 (2.93 to 3.99) | 3.55 (3.03 to 4.15) | 2.66 (2.11 to 3.36) |
| 40-49 | 9.37 (7 to 12.55) | 8.85 (7.23 to 10.83) | 9.96 (8.65 to 11.47) | 8.25 (7.11 to 9.58) | 6.9 (5.58 to 8.53) |
| 50-59 | 21.33 (16.08 to 28.29) | 21.34 (17.58 to 25.91) | 25.84 (22.54 to 29.62) | 14.87 (12.87 to 17.17) | 16.43 (13.42 to 20.12) |
| 60-64 | 40.6 (30.55 to 53.96) | 39.11 (32.19 to 47.52) | 46.23 (40.31 to 53.01) | 21.64 (18.69 to 25.05) | 26.73 (21.8 to 32.78) |
| **Sex (ref-female)** |  |  |  |  |  |
| Male | 2.49 (2.27 to 2.73) | 2.27 (2.13 to 2.42) | 2.26 (2.17 to 2.36) | 1.95 (1.85 to 2.06) | 1.89 (1.76 to 2.04) |
| **Foreign born (ref-US born)** |  |  |  |  |  |
| Foreign born | 3.09 (2.82 to 3.38) | 2.28 (2.14 to 2.42) | 2.26 (2.17 to 2.35) | 0.82 (0.78 to 0.87) | 0.93 (0.86 to 0.99) |
| **Race/ethnicity (ref-white)** |  |  |  |  |  |
| African American | 4.31 (3.56 to 5.21) | 3.84 (3.35 to 4.41) | 3.3 (3.02 to 3.61) | 2.48 (2.26 to 2.72) | 2.36 (2.08 to 2.67) |
| Latino | 4.76 (4.17 to 5.45) | 5.43 (4.93 to 5.98) | 4.31 (4.06 to 4.58) | 2.37 (2.22 to 2.54) | 2.19 (2 to 2.41) |
| Asian | 1.01 (0.83 to 1.24) | 1.07 (0.92 to 1.24) | 1.23 (1.12 to 1.34) | 0.69 (0.62 to 0.78) | 0.68 (0.58 to 0.79) |
| American Indian | 1.39 (0.62 to 3.13) | 2.81 (1.71 to 4.62) | 2.91 (2.27 to 3.73) | 4.07 (3.28 to 5.05) | 2.47 (1.77 to 3.44) |
| Native Hawaiian and Other Pacific Islander | 4.21 (2.59 to 6.85) | 9.19 (6.74 to 12.54) | 3.71 (2.99 to 4.61) | 2.97 (2.41 to 3.65) | 2.22 (1.6 to 3.09) |
| **Education (ref-HS)** |  |  |  |  |  |
| Bachelors, associate, or College | 0.42 (0.38 to 0.48) | 0.4 (0.37 to 0.44) | 0.53 (0.51 to 0.56) | 0.45 (0.42 to 0.48) | 0.45 (0.42 to 0.49) |
| Masters | 0.21 (0.15 to 0.28) | 0.16 (0.13 to 0.21) | 0.14 (0.12 to 0.16) | 0.14 (0.12 to 0.17) | 0.16 (0.12 to 0.20) |
| Professional | 0.37 (0.21 to 0.65) | 0.53 (0.37 to 0.75) | 0.57 (0.45 to 0.71) | 0.34 (0.25 to 0.46) | 0.30 (0.2 to 0.47) |
| Doctorate | 0.24 (0.12 to 0.46) | 0.18 (0.11 to 0.31) | 0.24 (0.18 to 0.32) | 0.11 (0.08 to 0.17) | 0.16 (0.09 to 0.28) |
| **Marital Status** |  |  |  |  |  |
| married | 1.92 (1.75 to 2.08) | 1.72 (1.64 to 1.85) | 1.56 (1.49 to 1.61) | 1.37 (1.30 to 1.45) | 1.64 (1.52 to 1.75) |

Note: estimates were derived from separate models for each characteristic in each wave.
